# Proteolytic Cleavage at Twin Arginine Residues Affects Structural and Functional Transitions of Lupin Seed 11S Storage Globulin

**DOI:** 10.1371/journal.pone.0117406

**Published:** 2015-02-06

**Authors:** Jessica Capraro, Fabio Sessa, Chiara Magni, Alessio Scarafoni, Elisa Maffioli, Gabriella Tedeschi, Ron R. D. Croy, Marcello Duranti

**Affiliations:** 1 Department of Food, Environmental and Nutritional Sciences, Università degli Studi di Milano, Milan, Italy; 2 Department of Veterinary Science and Public Health, Università degli Studi di Milano, Milan, Italy; 3 School of Biological and Biomedical Sciences, Durham University, Durham, United Kingdom; University of Wisconsin-Madison, UNITED STATES

## Abstract

The 11S storage globulin of white lupin seeds binds to a metal affinity chromatography matrix. Two unusual stretches of contiguous histidine residues, reminiscent of the multiple histidines forming metal binding motifs, at the C-terminal end of 11S globulin acidic chains were hypothesized as candidate elements responsible for the binding capacity. To prove this, the protein was incubated with a lupin seed endopeptidase previously shown to cleave at twin arginine motifs, recurrent in the sequence region of interest. Upon incubation with this enzyme, the loss of metal binding capacity paralleled that of the anti-his-tag reactive polypeptides. The recovered small proteolytic fragment was analyzed by mass spectrometry and N-terminal sequencing and found to correspond to the 24-mer region cleaved off at twin arginine residues and containing the natural his-tag-like region. Similarly, when lupin seeds were germinated for a few days, the his-tag containing 11S globulin chain was converted to a form devoid of such region, suggesting that this mechanism is a part of the natural degradatory process of the protein. The hypothesis that the ordered and controlled dismantling of storage proteins may generate peptide fragments with potential functional roles in plant ontogenesis is presented and discussed.

## Introduction

Despite a long history of investigations on legume seed storage proteins, our knowledge of the structural and functional features of this class of proteins is still incomplete and research continues to reveal new and intriguing properties. Among them, the frequent presence of twin and multiple arginine residues in the amino acid sequence of several storage proteins has been proposed as a cleavage-prone site for selective degradation [1]. A white lupin seed endopeptidase with specificity for peptide bonds containing double arginine (-R-R-) residues and active *in vitro* on selected storage proteins has been identified [1]. Although this activity is quite unusual in plants, in that it resembles the pro-protein conversion of some animal peptide hormones and secretory proteins [2], no attempts to investigate the potential structural and functional effects of this specific hydrolytic activity on seed storage proteins have been undertaken so far. Whether such specific cleavages take place in the plant kingdom, as they do in mammalian cells, leading to the liberation of biologically active peptides is not known yet, though the hypothesis of the role of adjacent basic residues as endopeptidase prominent recognition sites in seed proteins has already been put forward [3] and it is definitely intriguing. Several legume seed storage proteins of both the 7S and 11S globulin families contain twin arginine motifs in their amino acid sequences [1], and the existence of a -R-R- endopeptidase further supports this hypothesis.

In addition to such sequence peculiarity, various legume seed storage proteins, including soybean vicilin-like globulin [4], have been shown to be capable of binding metal ions. However, since the storage proteins are devoid of any apparent enzymatic activity, playing the alleged unique role of nitrogen and carbon skeleton source for the germinating plantlet, metals do not seem to be crucial for any specific biological activity nor for the assumption of the protein native conformation. Therefore, seed storage protein metal binding capacity has largely been neglected or simply considered an adventitious event, so far.

Another scarcely investigated aspect of structure/function relationships in the seed storage proteins is the description at molecular level of the detailed proteolytic modifications and the orderly dismantling of these proteins during seed germination [5, 6] and the possible roles of the transiently generated proteins/peptides. While a number of proteolytic enzymes have been shown to be involved in this phase of plant ontogenesis and their general mechanisms have been described [5, 7], the specific effects on the structural/functional properties of the protein substrates and their hydrolysis intermediate products have not received much investigation.

This work aims at filling some of these gaps and contributing to a better knowledge of these processes by using a white lupin seed legumin-like globulin as a model storage protein source.

## Materials and Methods

### General

Seeds of *Lupinus albus*, L., var. Multitalia were a kind gift from Agroservice Spa (San Severino Marche, MC, Italy). Anti-his-tag antibodies were purchased from GenScript (Piscataway, NJ, USA); antibodies to lupin 11S basic chain were prepared as described in ref. [8]. All other chemicals were reagent grade.

### Purification of lupin 11S globulin from mature dry lupin seeds

Lupin 11S globulin was purified as described previously [9]. Briefly, dry lupin seeds were dehulled and ground to a flour and then defatted in a Soxhlet apparatus. The defatted flour was extracted overnight at 4°C with 50 mM phosphate buffer pH 7.5 containing 0.5 M NaCl and a protease inhibitor cocktail (Sigma-Aldrich, Milan, Italy). The supernatant was subsequently desalted on a Sephadex G-50 column (GE Healthcare, Milan, Italy) equilibrated in 50 mM Tris-HCl, pH 7.5. The desalted extract was immediately fractionated on Whatman DE 52 DEAE cellulose column by increasing buffer NaCl concentration stepwise. The 11S globulin fraction was eluted at a NaCl concentration of 0.25M.

### Metal affinity chromatography of 11S globulin

The 11S globulin solution was loaded onto a preparative nickel affinity column (120 x 30 mm) (NiNTA-Agarose, QIAGEN, Milan, Italy) equilibrated with 50 mM Tris-HCl, pH 7.5, containing 0.5 M NaCl. The bound fraction was eluted with 0.25 M imidazole in the same buffer. For analytical purposes, 300 μg of 11S globulin sample were loaded onto a 5 mL NiNTA-HPLC column (IBA, Goettingen, Germany) equilibrated in 50 mM Tris-HCl pH 7.5 containing 0.5 M NaCl, at a flow rate of 1 mL/min and monitoring at 280 nm. After 12 minutes, the elution of his-rich peptides was carried out by a gradient of 0–25% (v/v) of 1 M imidazole for 2 minutes and maintaining 0.25 M imidazole concentration for further 10 minutes.

### RP-HPLC separation of NiNTA-bound peptide fragments

RP-HPLC of the fraction eluted by NiNTA using imidazole was carried out on a Symmetry300 C18 column (4.6 × 250 mm) (Waters, Milan, Italy), equilibrated with 0.1% (w/v) trifluoroacetic acid in water at pH 2.2. The elution of the peptide fractions, at a flow rate of 0.8 mL/min, was carried out by a continuous gradient of 0–75% (v/v) acetonitrile for 75 min and monitored at 220 nm.

### Lupin -R-R- endopeptidase and proteolytic activity assay

Lupin seed -R-R- endopeptidase was prepared using a combination of size exclusion, ion-exchange, affinity and metal affinity chromatography according to ref. [1]. For the proteolytic activity assay, the endopeptidase preparation was added to the 11S globulin solution in the ratio 1:20 (w/w). Samples were incubated at 37°C under stirring for 3, 6, 24, 48 and 72 hours in 100 mM Tris-HCl buffer pH 8.0 containing 0.15 M NaCl and 0.02% NaN_3_.

### Seed germination

Lupin seeds were vernalized at 4°C overnight and then germinated at 20°C for 24, 48, 72 and 96 hours. At the end of the incubation time, the seeds were dehulled, ground and the proteins extracted with SDS-PAGE denaturing solution containing 0.4% SDS and 2-mercaptoethanol in the ratio 1:20 w/v and immediately denatured at 100°C for 10 minutes. Extracts were centrifuged at 10,000 x *g* for 15 min and the proteins analyzed on SDS-PAGE as described in the next paragraph.

### SDS-PAGE and Western blotting

SDS-PAGE was carried out according to Laemmli [10] on 12% polyacrylamide gels. Protein samples were denatured in the presence of 2-mercaptoethanol by heating at 100°C for 10 min in the SDS/PAGE sample buffer and then loaded onto SDS polyacrylamide gels by using Mini-Protean 3 gel system (BioRad). After electrophoresis, bands were stained by Coomassie Blue G-250 (BioRad, Milan, Italy). The relative abundance and molecular mass of the polypeptides on scanned gels were determined by comparison with standard marker proteins (BioRaby using the Image-Master 1D Software (GE-Healthcare, Milan, Italy).

For immunodetection, the gels were transferred to nitrocellulose transfer membranes (Protran, Whatman, Dassel, Germany) by blotting [11] using a TE 77 Semidry transfer unit (GE-Healthcare, Milan, Italy). The membranes were blocked with 3% (w/v) gelatin for 1.5 h and washed three times for 10 min with 0.9% (w/v) gelatin, 0.05% Triton X-100 solution in PBS buffer (15 mM phosphate buffer pH 7.5 containing 0.15 M NaCl). Membranes were then soaked in PBS buffer containing rabbit anti-his-tag antibodies in the ratio 1:1,000 (v/v) or anti-lupin 11S basic chain in the ratio 1:1,500 for 1.5 h. To visualize the bands, blots were reacted with horseradish peroxidase conjugate with goat anti-rabbit antibodies (dilution 1:2,500; Bio-Rad) and after washing developed with H_2_O_2_ with 4-chloronaphthol as substrate.

### Mass spectrometry and amino acid N-terminal sequence analyses

For mass spectrometric analyses, RP-HPLC fractions were analyzed by MALDI-TOF mass spectrometry. Each sample was loaded onto a MALDI plate with a matrix of α-cyano-4-hydroxycinnamic acid. Mass spectrometric analysis was carried out on a Bruker Daltonics Reflex IV instrument (Bruker Daltonics, Milan, Italy) equipped with a nitrogen laser, operating in positive mode. Each spectrum was accumulated for at least 200 laser shots and Bruker peptide calibration standards were used for calibration.

Automated N-terminal amino acid sequence analysis was performed on a pulsed-liquid sequencer equipped with a PTH analyser (Procise model 491 Applied Biosystems, Foster City, CA).

## Results

### Metal affinity chromatography of lupin 11S globulin

**Fig 1:**
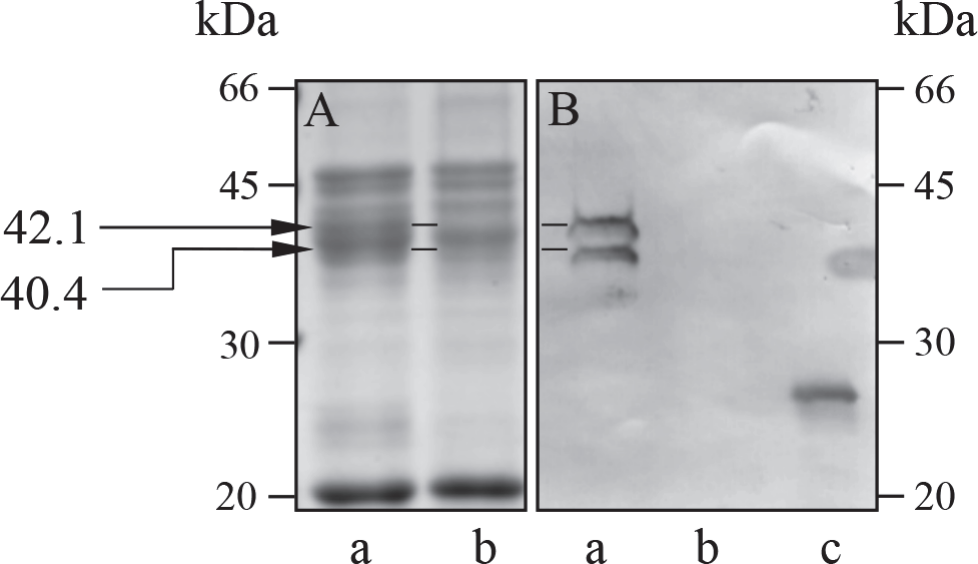
Coomassie blue-stained SDS-PAGE (A) and Western blot with anti-his-tag antibodies (B) of NiNTA-bound and unbound 11S globulin. The arrows indicate the reactive polypeptide chains of 42.1 and 40.4 kDa. Horizontal graphic lines between samples lanes indicate the bands correspondences. a: bound fraction; b: unbound fraction; c: his-tagged marker protein.

White lupin 11S globulin was purified from a mature dry seed protein extract, according to established protocols, based on anion exchange chromatography separation [9]. In the present study, the purified 11S globulin was also submitted to metal-affinity chromatography (NiNTA), as described under Methods. With this approach, a major bound fraction was recovered by addition of imidazole to the elution buffer. The bound fraction was submitted to SDS-PAGE analysis under reducing conditions (Fig. 1) and showed a group of polypeptides between 40 and 50 kDa, corresponding to the so called acidic chains, and a polypeptide chain of 20 kDa, referred to as the basic chain for its alkaline pI [9]. This polypeptide pattern is consistent with all seed 11S globulins, where the acidic and basic chains, are linked in a one-to-one ratio by one or more disulphide bridges, and are typically separated by electrophoresis under denaturing and reducing conditions [12].

**Fig 2:**
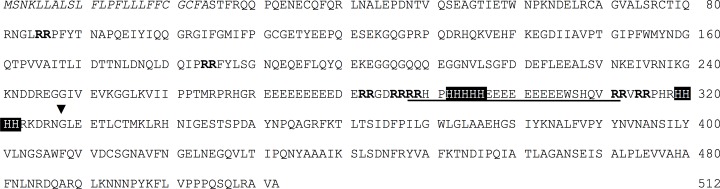
cDNA-deduced amino acid sequence of white lupin 11S globulin (UniProtKB: Q53I54). The signal peptide is indicated in italics. Twin arginine pairs are in bold. The 24-mer peptide generated by incubation of the 11S globulin with lupin -R-R- endoproteinase is underlined. Black blocks indicate the natural his-tag like regions (HHHH). ▼: conserved cleavage site between acidic and basic chains of 11S globulin family [3].

From the white lupin 11S globulin amino acid sequence available in the databases (UniProt KB: Q53I54, Fig. 2), we postulated that a C-terminal region of the acidic chain containing two proximal his-tag-like stretches of 5 and 4 contiguous histidine residues, H_292_-H_296_ and H_319_-H_322_ respectively, could be responsible for the protein’s metal binding capacity. By using an anti-his-tag antiserum, the reactive 11S globulin chains were shown on Western blots to be those of 42.1 and 40.4 kDa, indicated in Fig. 1 with arrows, while the bands around 50 kDa of the bound fraction and all the polypeptide chains of the unbound fraction did not react with the antiserum (Fig. 1). The presence of polypeptides lacking of his-tag-like regions in the SDS-PAGE pattern of NiNTA-bound 11S globulin is not surprising, being the lupin 11S globulin a randomly-assorted oligomer of heterogeneous subunits of different gene origin [12, 13].

### Effects of in vitro treatment of lupin 11S globulin with the endogenous -R-R- cleaving endopeptidase

**Fig 3:**
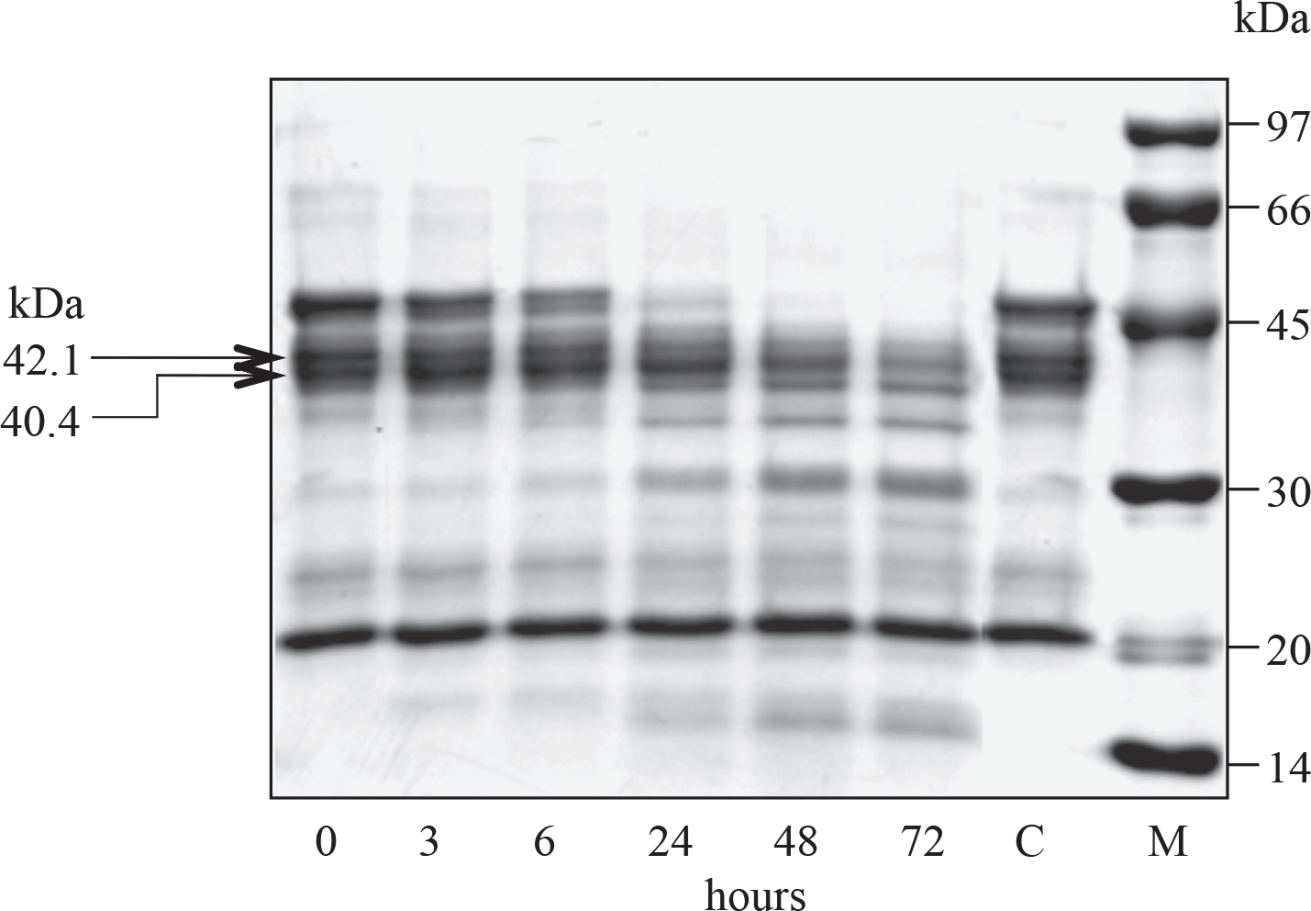
Coomassie blue-stained SDS-PAGE of NiNTA-binding 11S globulin treated with the -R-R- endopeptidase. Incubation times are indicated below each lane. M: marker proteins; C: control without enzyme upon 72 hours incubation. The arrows indicate the anti-his-tag antiserum reacting polypeptides.

To prove our hypothesis, the NiNTA-bound 11S globulin fraction was incubated with -R-R- endopeptidase prepared from mature dry lupin seeds. This enzyme was shown to preferentially cleave peptide bonds with at least two proximal arginine residues [1]. In the his-rich C-terminal sequence of the acidic chains, there are various twin arginine motifs (Fig. 2). SDS-PAGE analyses (Fig. 3) of the NiNTA-bound 11S globulin after various times of incubation with the -R-R- endopeptidase showed the progressive disappearance of the largest-sized acidic polypeptides, including those reactive with the anti-his-tag antiserum (cfr. Fig. 1), and the concomitant increase of various smaller chains. In particular, the polypeptides of 50 kDa were completely affected by proteolysis (Fig. 3), because of the presence of twin arginine pairs (Fig. 2). The basic polypeptides remained unaffected by the hydrolytic activity, both because they reside inside the native structure of legumin [7, 12] and because they are devoid of any -R-R- motif in the sequence.

**Fig 4:**
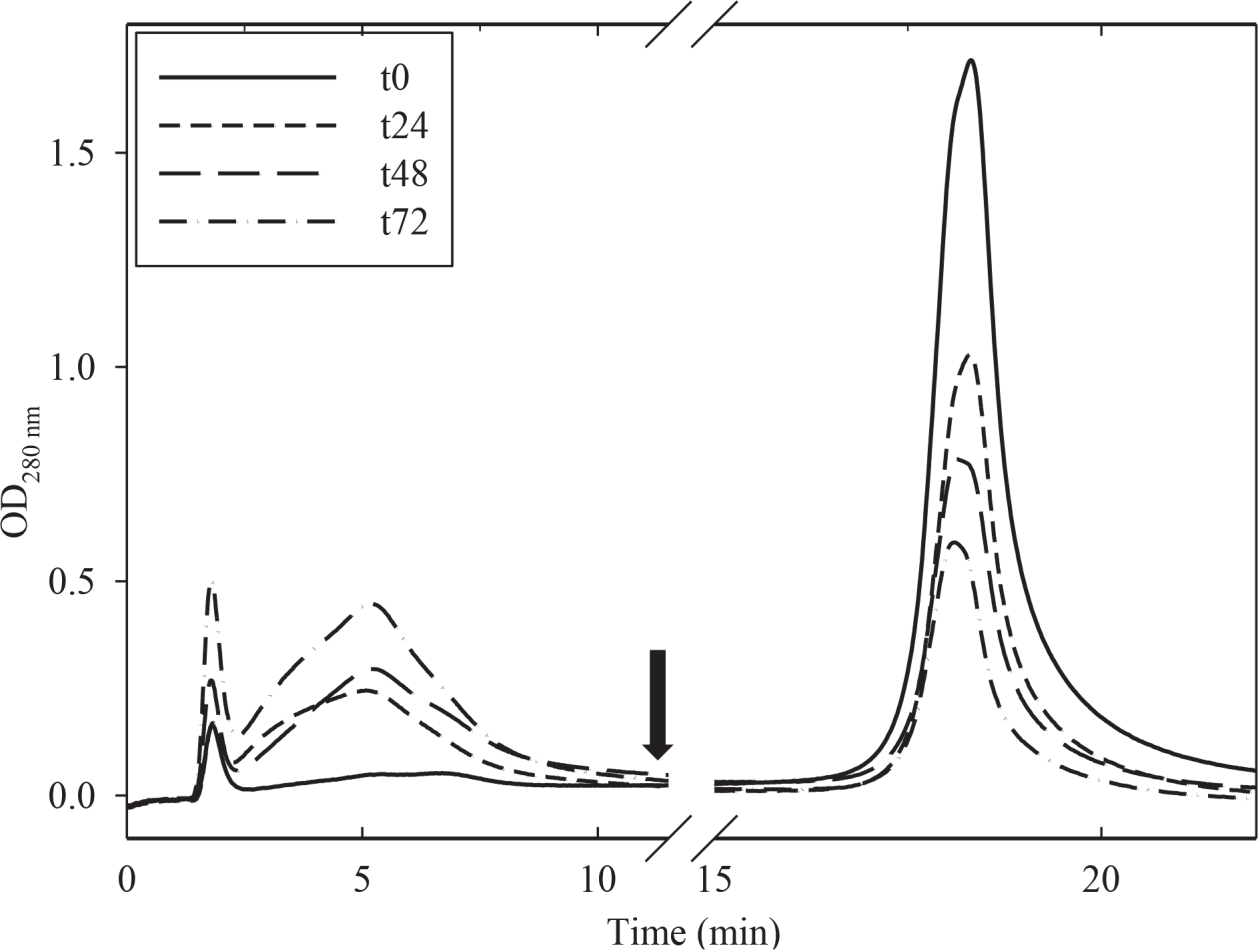
NiNTA separation of purified lupin 11S globulin incubated with the -R-R- endopeptidase. The incubation times indicated in the inset are expressed as hours. The arrow indicates addition of 250 mM imidazole to the elution buffer. The spectra were recorded at 280 nm.

When the endopeptidase-treated samples were separated on NiNTA, there was a progressive decrease in the bound fraction with a concomitant increase in the unretained fraction, as monitored at 280 nm in the chromatogram of Fig. 4, thus suggesting the proteolytic removal of both natural his-tag-like regions at the C-terminus of the acidic chains.

**Fig 5:**
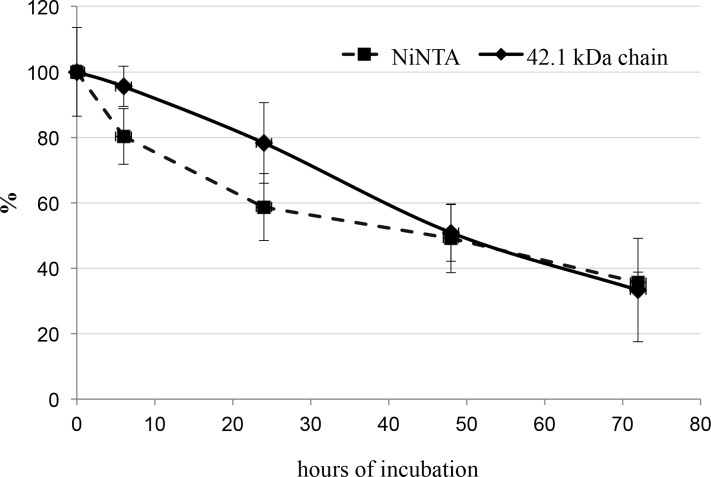
Comparative quantitative analysis of NiNTA-binding fraction of lupin 11S globulin and the 42.1 kDa polypeptide chain. The proteins were incubated with -R-R- cleaving endopeptidase for the indicated times. NiNTA-binding fraction was calculated as area under the curve in the chromatogram. The 42.1 kDa polypeptide band in SDS-PAGE was quantified by densitometric analysis, Data are expressed as percent of the value at zero time (t_0_) and represent the average of 3 separate replica. Vertical bars indicate standard deviation of the mean.

Densitometric data, obtained by scanning the most representative his-tag band of 42.1 kDa in SDS-PAGE analyses of the treated samples during the course of incubation (Fig. 3), were then compared with the relative decrease in NiNTA-bound 11S globulin form in Fig. 5. Data were expressed as average percent decrease from t_0_ sample (control) to make the two graphs comparable. Slopes were similar, thus suggesting a correlation between removal of the his-tag-like peptide region and loss of NiNTA-binding capacity of the lupin 11S globulin.

**Fig 6:**
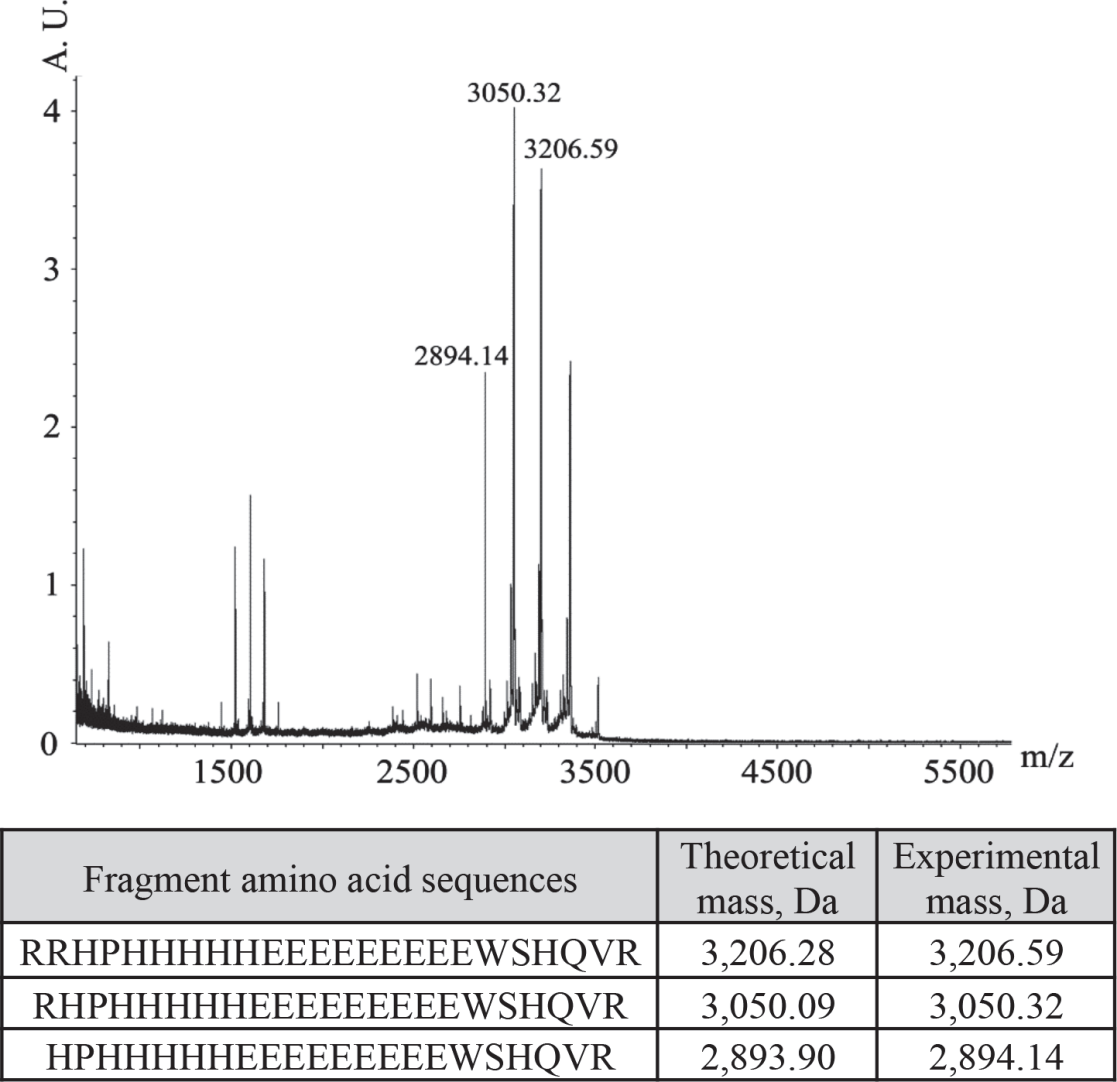
MALDI-TOF spectra of the main his-rich peptide fragment. The table reports the fragments identified by N-terminal analyses. The position of the peptides along the 11S globulin amino acid sequence (UniProtKB Q53I54) is 289–311, 288–311 and 287–311, respectively. To locate these fragments in the DNA-deduced amino acid sequence of lupin 11S globulin refer to Fig. 2.

To identify the cleaved peptide fragment, a preparation of extensively endopeptidase-digested 11S globulin was loaded onto a NiNTA column. The retained fraction was eluted by using imidazole and submitted to RP-HPLC and gave rise to two peaks (not shown). The peaks were collected and analyzed by MALDI-TOF spectrometry. Only the major peak gave reliable molecular masses around 3,000 Dalton (Fig. 6). These masses perfectly matched the theoretical masses of the putative fragments at R_288_, R_289_ and H_290_. In parallel, N-terminal sequencing of the peptide confirmed the cleavages in the indicated positions starting from RRHP (Fig. 6). This latter position corresponded to the 24-mer region underlined in the full length 11S globulin sequence of Fig. 2. Therefore, *in vitro* enzymatic cleavage effectively removed the metal binding, his-tag-like regions from the 11S globulin and provided details on the nature of the cleaved peptide and cleavage site. From our findings, the removed peptide appears to be necessary and sufficient to confer metal binding capacity to the intact protein.

### Effects of lupin seed early germination on the covalent processing of the 11S globulin

**Fig 7:**
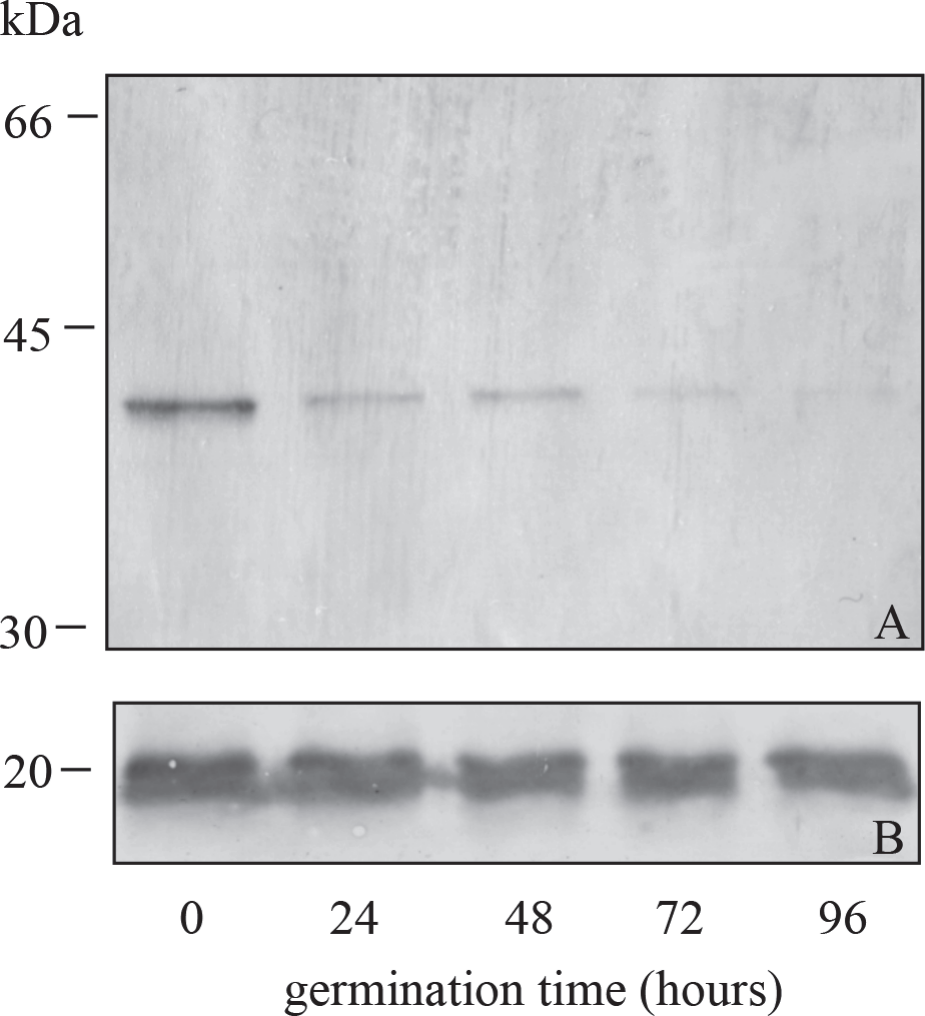
Western blot analyses of total proteins extract from lupin germinated for the indicated times. A. Detection with anti-his tag. B. Detection with anti-basic polypeptide antisera.

Lupin seeds were submitted to germination, as described under Methods. From a previous work [14], it was known that degradation of the lupin 11S globulin is at its maximum about 6–7 days into germination. Therefore, we focused on the initial steps of germination, where more subtle modifications to the storage proteins might be expected. To prevent additional *in vitro* degradation, proteins were extracted directly from germinating cotyledons with sample denaturing buffer, at 100°C and then analyzed by SDS-PAGE under reducing conditions. The separated polypeptides were blotted onto nitrocellulose membrane and reacted with anti-his-tag antibodies. The results are shown in Fig. 7A. Immediately obvious from this analysis was the dramatic decrease in the 11S polypeptide acidic chain around 40 kDa which reacted with the anti-his-tag antibodies within the first 24 hours of germination and then further decreased and became almost undetectable at 72–96 hours, under our experimental conditions. In parallel, in order to check the overall stability of the 11S globulin, a polyclonal antibody against the N-terminal region of the 11S globulin basic chain was used with an identical blotted SDS-PAGE separation of the germinating lupin seed (Fig. 7B). The basic polypeptide was almost unaffected. These findings showed a quantitative removal of the his-tag-like containing polypeptide during germination, similar to that observed following *in vitro* incubation of the lupin 11S globulin with the -R-R- endopeptidase, though with expectedly different dynamics. It is worth noting that one of the two anti-his-tag reacting polypeptides present in the mature quiescent seeds (Fig. 1B) rapidly disappeared at the onset of germination, with only one polypeptide being detectable since the very first phases of germination (Fig. 7A). By and large, during germination the removal of the natural his-tag-like region, prior to the complete globulin degradation, was responsible for the liberation of the metal binding peptide/s of lupin 11S globulin as a part of the natural process of storage protein mobilization and degradation, as discussed in ref. [6].

## Discussion

The present study focuses on subtle, but relevant changes to the covalent structure of a legume seed storage protein induced by a novel protease activity and affecting its functionality. We have drawn on both *in vitro* and *in vivo* approaches to study the phenomenon and to infer the potential biological significance of the results. The site-specific proteolytic cleavage at twin arginine residues motifs by the lupin -R-R- cleaving endopeptidase removed the natural his-tag-like region in the lupin 11S globulin acidic chain, thus causing the loss of the metal binding capacity of this storage protein. An equivalent proteolytic event, occurring during early germination of lupin seeds, had a similar effect, thus liberating the natural metal chelating domain from the C-terminal end of the 11S globulin acidic chain.

**Fig 8:**
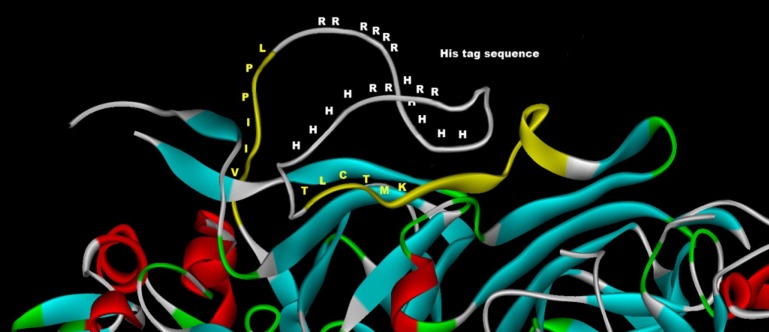
Detail of a predictive 3D model showing the surface-located his-tag-like sequence of lupin 11S globulin and the arginine peptide bonds cleaved by the -R-R- endopeptidase (not to scale). The predictive model was built using rapeseed cruciferin A (PDB: 3KGL) as a template. The sequence of the variable region IV of cruciferin has been replaced with the corresponding sequence of lupin 11S globulin.

The release of the his-tag-like region promoted by the -R-R- specific endopeptidase and the abundance of acidic and basic amino acids and histidine residues in the cleaved fragment suggest that this region and the target peptide bonds lie in an exposed peptide loop near the surface of the 11S globulin. The comprehensive structural comparisons of 11S globulin 3D structures published by Tandang-Silvas et al. [15] has allowed us to confirm this hypothesis. A Clustal Omega alignment (http://www.clustal.org/omega/) was carried out on 9 polypeptide sequences, namely lupin 11S globulin (UniProtKB:Q53I54), pea legumin (UniProtKB:P02857), cruciferin A chain (PDB: 3KGL) and the 6 cruciferin sequences from 3KGL which were fixed in the crystallographic structure [15]. The disorganized sequences with no spatial positions assigned were removed from 3KGL using Discovery Studio Visualizer v1.7 (*Accelrys*). The results of this alignment (not presented) were highly comparable with that of Tandang-Silvas et al. [15], with the lupin 11S globulin included, which clearly showed the his-tag sequence was located in variable region IV towards the C-terminal of the acidic polypeptide. This sequence could be mapped on the surface of the protein, as shown by the predictive model prepared using the cruciferin structure as template, in which its variable region IV has been substituted with the corresponding variable region of lupin 11S globulin. An enlarged view of the predicted 3D model is shown in Fig. 8.

In a previous work [1], the hypothesis of a functional role for the presence of cleavage-prone sequence motifs in the legume and non-legume storage proteins, aimed at generating biologically functional fragments, has already been proposed. For example, the activation during lupin seed germination of a cryptic lectin-like and anti-fungal lupin 7S globulin fragment, termed BLAD [16, 17] was mimicked *in vitro* with the -R-R- cleaving endopeptidase [1]. No further molecular studies on such processes have been undertaken yet. In this work, specific -R-R- cleavages leading to the removal of the his-tag-like containing peptides was actually monitored. The limited cleavage was shown to abolish the whole protein metal binding capacity.

The presence of independently acting regions of seed proteins has already, though not frequently, been documented, such as the case of antimicrobial peptides originating from seed storage protein hydrolysis, with activity against fungi and insects [18]. In soybean seeds, an extension peptide belonging to phytoferritin with a serine protease-like activity acted as a conversion enzyme to increase the rate of ferritin iron release [19]. In this case, no cleavage specificity for dibasic amino acid motifs has been described. Conversely, mammalian pro-protein maturation to various hormone peptides, occurring at internal dibasic amino acid sites [2, 20] with the involvement of endoproteinases, such as kexin and pro-protein convertases, has frequently been reported [21]. In the plant kingdom, although the presence of kexin-like, pyrolysin-like and subtilase activities effective on dibasic amino acid sites and involved in the maturation or degradation of some plant peptide hormones has been documented [22], examples of protein maturation and activation through specific proteolytic events are quite rare. In this respect, the processing of pro-legumin precursor during its synthesis and maturation at a highly conserved -N-G- site is a well-known example [23], but it does not involve dibasic amino acids. This cleavage was shown to trigger the trimer to hexamer structural transition occurring in the conversion of pro-legumin to mature legumin [6, 13].

Many seed storage proteins contain from 1 up to 12 twin arginine motifs [1], lupin proteins being among the richest ones. These sites possibly act as pre-determined cleavage positions and, if so, it is arguable that the liberated fragments may play functional role in the seed. The rapid alkalinization factor of mouse-ear cress (RALF1; UniProtKB: Q9SRY3_ARATH) is probably activated by a kexin-like convertase specific for a dibasic site [24] and the antimicrobial polypeptide tomato defensin also has a stretch of multiple arginine residues (UniprotKB: B1N678_SOLLC), but no data on their role either in the processing or activation of its defense activity are available [25]. Other anti-microbial polypeptides of about 20 kDa have been isolated from pea seeds [26], but no information on their primary structure nor on their derivation from larger precursors are available, thus preventing any conclusion to be drawn.

This work did not aim to measure any physiologically-relevant activity of the liberated peptides; however their metal binding capacity speaks in favour of a metal mobilization/chelation role. Indeed, although little is known about the potentially limiting role of mineral nutrients in early metabolic and developmental processes during germination, our findings support an involvement of these metal-containing peptides in the reallocation of several ions from germinating cotyledons to root and hypocotyl tissues [27]. A defense role of the his-tag-like containing peptides depending on their metal chelation capacity, as has been observed for animal proteins like calprotectin [28], lactoferrin [29] and others, cannot be excluded. Interestingly, the cattle tick antimicrobial activity cys/his rich, Cu^++^-binding polypeptide, termed microplusin, has unequivocally been attributed to its copper depletion mode of action [30]. The present findings conform to the previous identification of inhibitory activities buried into maturing storage protein precursors, namely *Cucurbita maxima* provicilin [31] and *Heliantus annuus* proalbumin [32].

This work, by showing the specific removal of the his-tag-like region of germinating lupin seed 11S globulin prior to globulin collapse, further supports the former hypotheses on an orderly deconstruction of storage proteins during germination rather than an extensive, random proteolytic digestion [5, 6] and also add some insight on the potential physiological role of the transiently liberated peptide fragments.

The extent to which storage proteins lose intact peptides and whether the removed peptides have biological activities useful for the plants early growth are questions alluded to here and are worthy of further study. Nonetheless, the present findings on a natural metal chelator originating from a lupin storage globulin, through a mammalian-like specific proteolytic event, open up new perspectives in the identification of cryptic bioactivities related to seed ontogenesis and protection.
